# Cardiac valve prosthesis choice for left-sided infective endocarditis in Medicare patients

**DOI:** 10.1016/j.xjon.2025.09.050

**Published:** 2025-10-24

**Authors:** Waseem Lutfi, Ziwei Pan, Madison A. Grasty, Nicolas J. Goel, Michael A. Catalano, Alexandra E. Sperry, Jonathan Szeto, Pablo Kurzan, Kendall M. Lawrence, Nimesh D. Desai, Wilson Y. Szeto, John H. Holmes, Chase R. Brown

**Affiliations:** a Division of Cardiovascular Surgery, University of Pennsylvania, Philadelphia, Pa; b Perelman School of Medicine, University of Pennsylvania, Philadelphia, Pa; c Department of Biostatistics, Epidemiology, and Informatics, University of Pennsylvania, Philadelphia, Pa

**Keywords:** infective endocarditis, mechanical valves, bioprosthetic valves, aortic valve replacement, mitral valve replacement, overall survival

## Abstract

**Objective:**

To compare the outcomes between bioprosthetic and mechanical prosthesis choice for aortic valve and mitral valve infective endocarditis (IE) in patients >65 years old.

**Methods:**

Medicare Provider Analysis and Review files from 2011 to 2019 were queried for adult patients >65 years old with de novo IE who underwent isolated bioprosthetic aortic valve replacement (bAVR), mechanical aortic valve replacement (mAVR), bioprosthetic mitral valve replacement (bMVR), or mechanical mitral valve replacement (mMVR). Patients with preoperative ischemic or hemorrhagic stroke were excluded. Analyses were conducted separately for the aortic and mitral position: bAVR versus mAVR, and bMVR versus mMVR. Propensity score matching was used to account for measured confounders. The primary outcome was 5-year overall survival analyzed using restricted mean survival time; secondary outcomes were the 5-year cumulative incidences of valve reoperation, heart failure readmission, recurrent IE, bleeding, and ischemic stroke analyzed using Fine-Gray regression with death as a competing risk.

**Results:**

Matching yielded 330 patients in each AVR group and 250 patients in each MVR group. Five-year survival favored bAVR over mAVR (66.1% vs 56.7%, RMST: 3.96 years vs 3.46 years, *P* = .001), whereas 5-year survival was similar for bMVR versus mMVR (53.1% vs 53.1%, RMST: 3.50 years vs 3.35 years, *P* = .451). There were no significant differences in the cumulative incidences of secondary outcomes.

**Conclusions:**

This analysis of patients >65 years old with left-sided IE demonstrated that mechanical valves were associated with worse early mortality, no 5-year survival advantage over bioprosthetic valves, and provide no benefit in this population.

## Graphical Abstract

**Figure:**
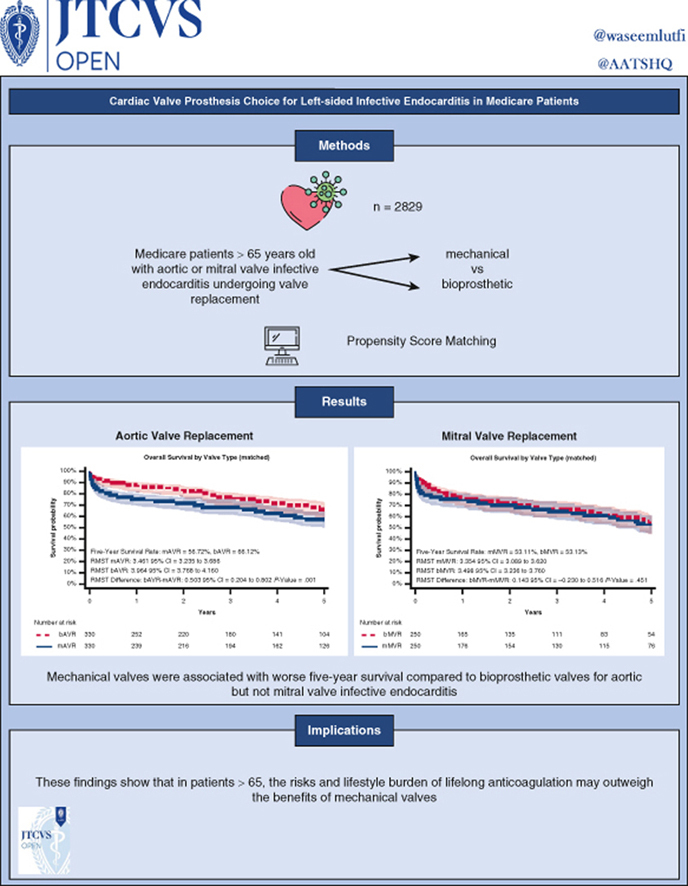

**Figure:**
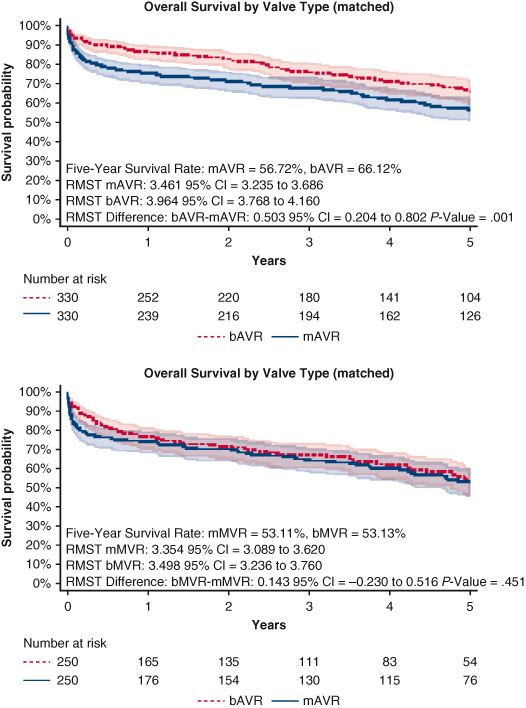
Propensity score−matched 5-year overall survival by prosthesis choice for AVR and MVR.

**Central Message**

Patients >65 years old with left-sided infective endocarditis can safely undergo valve replacement, and there is no survival benefit associated with mechanical valves over bioprosthetic valves.

**Perspective**

There is a paucity of published data comparing valve choice in patients >65 years old with left-sided infective endocarditis. Contrary to studies in middle-aged patients showing a survival benefit associated with mechanical valve replacement, these findings show that in patients >65 years, the risks and lifestyle burden of lifelong anticoagulation may outweigh the benefits of mechanical valves.

When choosing between a mechanical or bioprosthetic valve prosthesis for left-sided infective endocarditis (IE), surgeons must weigh patient preference and ability to adhere to strict anticoagulation dosing, valve durability in terms of the risks of reinfection or reoperation, risk of stroke and hemorrhage, as well as long-term survival. For younger patients <65 years old with IE, several large cohort studies have demonstrated that mechanical valves are associated with lower long-term mortality and reoperation compared with bioprosthetic valves.^1^, ^2^, ^3^, ^4^, ^5^ However, among patients >65 years old with left-sided IE, the optimal prosthetic valve choice for surgical valve replacement is less clear.

Very few studies have directly evaluated prosthesis choice in patients >65 years old undergoing valve replacement for left-sided IE and are limited to small cohorts underpowered to provide inferable conclusions.^4^^,^^6^ Reflective of the lack of direct head-to-head comparisons of mechanical versus bioprosthetic valves for left-sided IE in patients >65 years, current guidelines recommend that prosthesis choice should be determined by normal age criteria, which typically prefers bioprosthetic valve choice in this age cohort, a Grade 2A recommendation.^7^^,^^8^ Given the limited data in patients >65 years, in this study, we compared long-term outcomes between bioprosthetic and mechanical valve choice for aortic valve and mitral valve IE in patients >65 years old using Medicare data.

## Methods

### Study Population and Data Source

**Figure 1:**
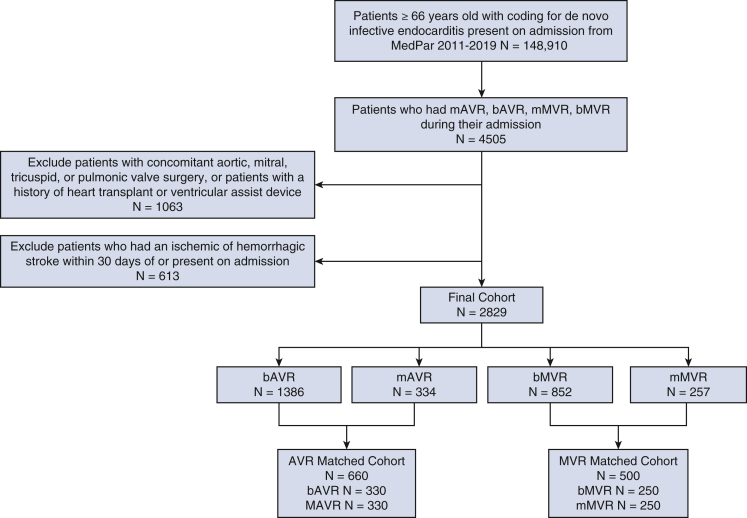
Consolidated Standards of Reporting Trials diagram. *MEDPAR*, Medicare Provider Analysis and Review; *mAVR*, mechanical aortic valve replacement; *bAVR*, bioprosthetic aortic valve replacement; *mMVR*, mechanical mitral valve replacement; *bMVR*, bioprosthetic mitral valve replacement; *AVR*, aortic valve replacement; *MVR*, mitral valve replacement.

Medicare MEDPAR files were available from 2009 to 2020; however, our cohort was restricted to the years 2011 to 2019 and patient ages ≥66 years old to allow characterization of patient comorbidities from Medicare claims before valve replacement surgery and at least 1 calendar year of follow-up. Thus, MEDPAR files from 2011 to 2019 were queried for all adult patients ≥66 years old who had de novo IE (meaning a first-time diagnosis of IE) that was present on admission then underwent isolated bioprosthetic aortic valve replacement (bAVR), mechanical aortic valve replacement (mAVR), bioprosthetic mitral valve replacement (bMVR) or mechanical mitral valve replacement (mMVR). *International Classification of Diseases* (ICD) *Ninth* (-9) and *Tenth* (-10) *Revisions*, Procedure Coding System codes from inpatient MEDPAR claims were used to select for aortic valve replacement (AVR) and mitral valve replacement (MVR) procedures, whereas Clinical Modification diagnosis codes were used to define IE. Patients were excluded if they had a history of IE found in any previous claims, any concomitant valve or aortic surgery (ie, isolated AVR or isolated MVR only), previous heart transplant or ventricular assist device, or an ischemic or hemorrhagic stroke within 30 days of admission or that was present on admission. We excluded preoperative ischemic and hemorrhagic strokes within 30 days to reduce bias by indication because anticoagulation requirements for mechanical valves and the risk of cerebral hemorrhage may have precluded mechanical valve choice. Figure 1 shows the selection criteria consort diagram, and Table E1 shows the ICD coding schema used in this study. Per the University of Pennsylvania Institutional Review Board policy, this study was exempt from institutional review board review, and patient informed written consent was waived because this study used deidentified claims data.

### Study Design and Outcomes

A retrospective cohort study design was conducted with the exposure of valve prosthesis type. Two parallel but separate comparisons were made: comparing bAVR versus mAVR, and bMVR versus mMVR. The primary outcome was overall survival up to 5 years. Secondary outcomes included admission for recurrent endocarditis, valve reintervention, readmission for congestive heart failure, ischemic stroke, and a composite bleeding outcome of gastrointestinal bleeding, cerebral hemorrhage, and other bleeding; all secondary outcomes required a present-on-admission indicator with the ICD code. Valve reoperation was defined for AVR as a repeat AVR, transcatheter aortic valve replacement, or open thoracic aortic procedure; for MVR valve reoperation was defined as a repeat MVR. Covariates of interest included patient age, sex, Elixhauser comorbidities and index score,^9^ concomitant coronary artery bypass graft (CABG), percutaneous coronary intervention (PCI) within 3 months before valve replacement, infectious micro-organisms, and the following risk factors for IE: a previous prosthetic valve, an implantable cardiac device, and opioid use disorder. ICD-9 and ICD-10 Clinical Modification codes as well as Procedure Coding System codes that were used to define covariates (not including Elixhauser coding) are shown in Table E1. Elixhauser comorbidities were ascertained through all previous MEDPAR claims before AVR or MVR.

### Statistical Analysis

Baseline cohort characteristics were compared using the Student *t* tests for continuous variables and χ^2^ tests for categorical variables. One-to-one (1:1) propensity score matching was used to match for all previously mentioned covariates between comparison groups (bAVR vs mAVR and bMVR vs mMVR). Propensity scores were generated using the MatchIt R package (version 4.5.5)^10^; logistic regression modeling to generate propensity scores included age, sex, each individual Elixhauser comorbidity variables (31 total) and overall index score,^9^ concomitant CABG, PCI within 3 months before valve replacement, infectious micro-organisms, a previous prosthetic valve, an implantable cardiac device, and opioid use disorder. 1:1 propensity-score matching was performed using greedy nearest neighbor matching without replacement with a caliper width of 0.1. Love plots demonstrating standardized mean differences of covariates included in the propensity score model before and after matching were used to assess balance of covariates with a 10% mean standardized difference cutoff for covariate balance acceptability. Kaplan-Meier methods with restricted mean survival time (RMST) differences were used to compare 5-year overall survival between comparison groups. The RMST represents the area under the Kaplan-Meier survival curves and is interpreted as the average time until an event occurs during a defined time period; in this study from time 0 to 5 years. The RMST is a powerful statistic that is easy to interpret with fewer assumptions than other time-to-event summary statistics such as hazards ratios generated by the Cox proportional hazards regression.^11^ The *strmst2* command in Stata was used to generate RMST values and differences.^12^ Because of death as a competing risk, secondary outcomes were compared using Fine-Gray subdistribution hazard models and displayed using cumulative incidence functions up to 5 years.^13^ Subhazard ratios with 95% confidence intervals (95% CI) were used for exposure effect estimates.

To assess for any potential residual unmeasured confounding after propensity score matching, we used the 2 falsification end points of urinary tract infection (UTI) and hip fractures comparing the cumulative incidences of each on Fine-Gray analysis with death as a competing risk. These end points have been used in previous studies.^14^

All tests were 2-sided, and an alpha of 5% was used as the cutoff for statistical inference. Statistical software used included R Statistical Software (version 4.2.1; R Core Team 2023), and Stata MP, version 18 (StataCorp LLC).

### Subgroup Analyses

To examine potential residual confounding attributable to preoperative anticoagulation use influencing AVR and MVR prosthesis choice, Medicare Part D files available from 2016 to 2019 were used to identify patients who were preoperatively prescribed and filled the following anticoagulation prescriptions within 6 months before surgery: warfarin, enoxaparin, apixaban, rivaroxaban, fondaparinux, edoxaban, betrixaban, or dabigatran. Our department had previously purchased Part D data for the years 2016 to 2019 and thus those were the only years available for our study. We compared the rates of mechanical versus bioprosthetic valve choice among patients with Medicare Part D data who were on preoperative anticoagulation and those who were not. Similarly, we also evaluated the rates of mechanical versus bioprosthetic valves in patients with a preoperative ischemic or hemorrhagic stroke that were excluded from our primary analysis.

To evaluate the impact of age on survival outcomes, we stratified our cohort into 2 age subgroups: patients aged 65 to 74 years and patients aged ≥75 years. We then repeated our propensity score matching algorithms within each subgroup and compared our primary outcome of 5-year overall survival.

### Post Hoc Logistic Regression Identifying Predictors of 30-Day and 90-Day Mortality

After conducting our primary analysis, it was noted that mechanical valves were associated with worse early mortality. We thus endeavored to identify predictors of 30-day and 90-day mortality among our matched cohorts. Forward stepwise multivariable logistic regression analysis was used to identify predictors of 30-day and 90-day mortality using the automated *stepwise* command in Stata with a *P* value of entry of .100 and a *P* value of removal of 0 = .150. Covariates included valve choice, and all covariates included in our propensity score generation.

## Results

### Cohort Characteristics

After patient selection, 2829 patients were included for analysis; the average age was 73.4 years, 67.4% of patients were male, 85.0% were White, 8.3% were Black, and 6.7% were other races. During this study period, most patients had biologic valves implanted. In total, 1386 underwent bAVR, 334 mAVR, 852 bMVR, and 257 mMVR; respectively, the mean ages were 73.6 years versus 73.0 years (*P* = .08), and 73.5 years versus 72.7 years (*P* = .05) and mean Elixhauser scores were 7.9 versus 7.1 (*P* < .001) and 7.7 versus 7.5 (*P* = .25), indicating that mechanical valve patients were younger and with fewer comorbidities while bioprosthetic valve patients were older with higher comorbidity burden. Rates of CABG were 12.2% for AVR and 9.9% for MVR and rates of perioperative PCI were 1.3% for AVR and 0.4% for MVR. Table E2 demonstrates the cohort covariate characteristics by AVR and by MVR type before propensity score matching.

### Overall Survival Before Propensity Score Matching

**Figure E1:**
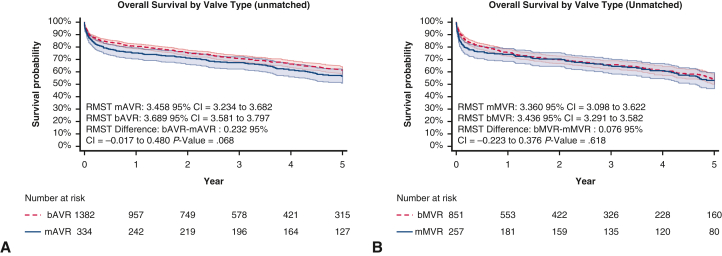
Unmatched 5-year overall survival by prosthesis choice for (A) AVR and (B) MVR with 95% CI. *AVR*, Aortic valve replacement; *MVR*, mitral valve replacement; *CI*, confidence interval; *RMST*, restricted mean survival time; *mAVR*, mechanical aortic valve replacement; *bAVR*, bioprosthetic aortic valve replacement; *mMVR*, mechanical mitral valve replacement; *bMVR*, bioprosthetic mitral valve replacement.

Figure E1 shows the Kaplan-Meier survival curves by AVR and MVR types before matching. Patients with mechanical valves had a shorter 5-year RMST compared with bioprosthetic valves with mAVR at 3.46 years (95% CI, 3.24-3.69) versus bAVR at 3.69 years (95% CI, 3.58-3.80), and mMVR at 3.36 years (95% CI, 3.10-3.62) versus bMVR at 3.44 years (95% CI, 3.29-3.58). RMST differences are shown in Figure E1.

### mAVR Versus bAVR Matching and Outcomes

**Figure 2:**
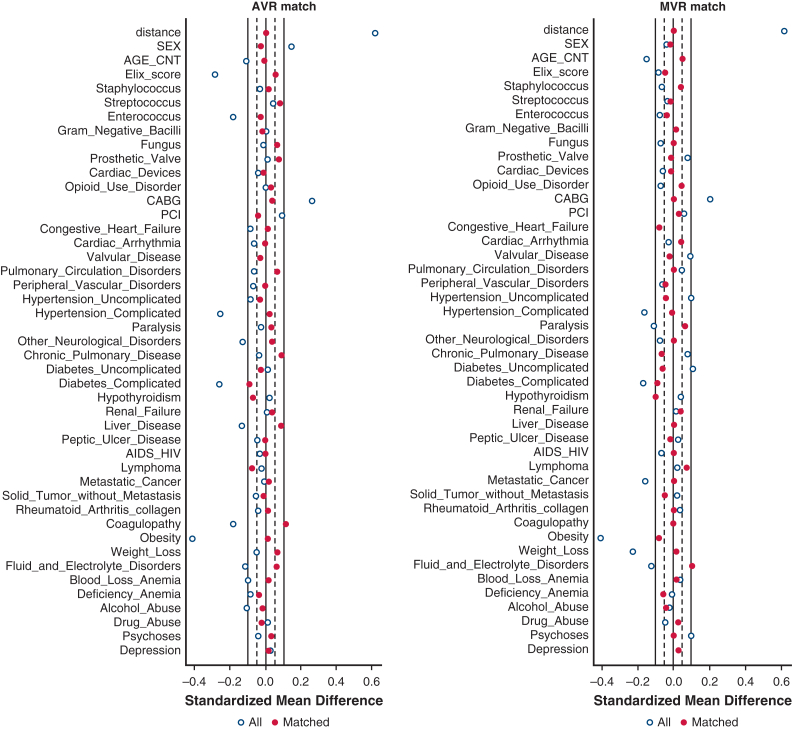
Propensity-score matching: standardized mean difference love plots. *AVR*, Aortic valve replacement; *MVR*, mitral valve replacement; *CABG*, coronary artery bypass grafting; *PCI*, percutaneous coronary intervention.

**Figure 3:**
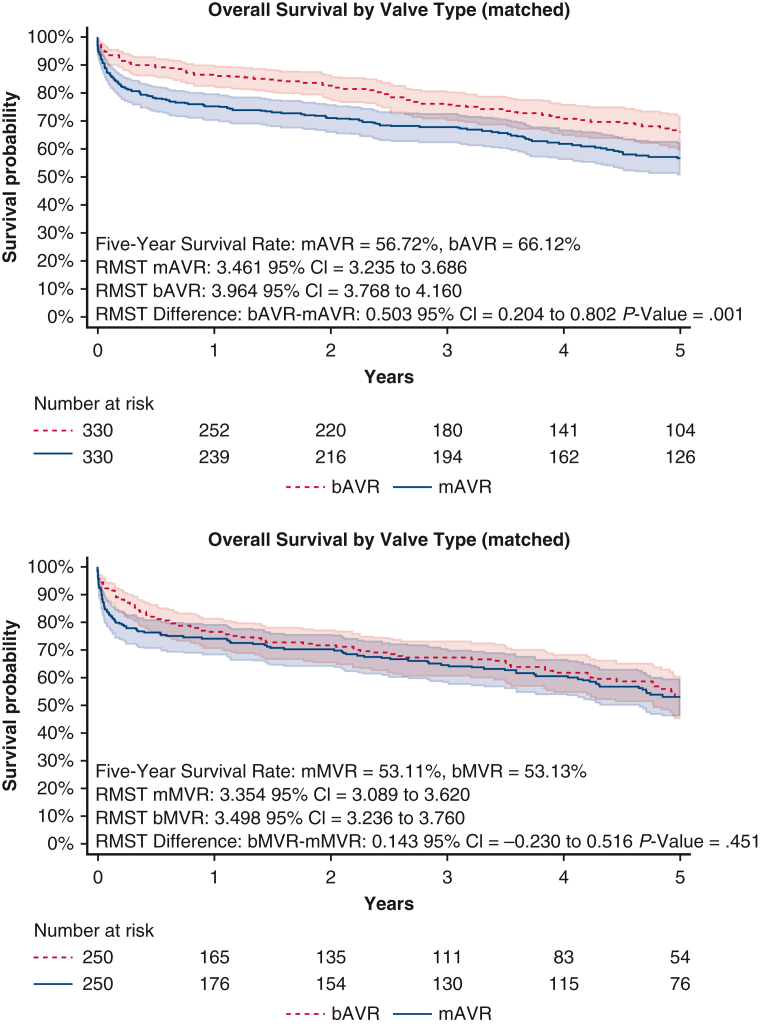
Matched 5-year overall survival by prosthesis choice for AVR and MVR with 95% CI. *AVR*, Aortic valve replacement; *MVR*, mitral valve replacement; *CI*, confidence interval; *mAVR*, mechanical aortic valve replacement; *bAVR*, bioprosthetic aortic valve replacement; *RMST*, restricted mean survival time; *mMVR*, mechanical mitral valve replacement; *bMVR*, bioprosthetic mitral valve replacement.

Figure 2 shows the standardized mean differences among matched covariates, indicating a well-balanced match. Matching yielded 330 patients in each group. Table 1 shows cohort characteristics after matching. Compared with bAVR, mAVR was associated with a worse 5-year overall survival at 56.7% for mAVR versus 66.1% for bAVR (Figure 3) with RMST at 3.46 years for mAVR and 3.96 years for bAVR with a RMST difference of 0.50 years (95% CI, 0.204-0.802, *P* = .001).

**Table 1:** Matched cohort characteristics

Characteristic	mAVR	bAVR	SMD	mMVR	bMVR	SMD
N	330	330		250	250	
Age, y, mean (SD)	73.01 (5.62)	73.05 (5.83)	−0.007	72.78 (5.16)	72.53 (5.06)	0.049
Sex			−0.027			−0.016
Male	233 (70.61%)	229 (69.39%)		139 (55.60%)	137 (54.80%)	
Female	97 (29.39%)	101 (30.61%)		111 (44.40%)	113 (45.20%)	
Elixhauser score, mean (SD)	7.11 (2.95)	6.94 (2.90)	0.057	7.44 (3.01)	7.59 (3.07)	−0.048
Coronary artery bypass graft	67 (20.30%)	62 (18.79%)	0.037	35 (14.00%)	35 (14.00%)	0.000
Percutaneous coronary intervention	6 (1.82%)	8 (2.43%)	−0.040	0 (0.00%)	2 (0.80%)	0.032
Prosthetic valve	70 (21.21%)	60 (18.18%)	0.075	43 (17.20%)	44 (17.60%)	−0.011
Cardiac device	27 (8.18%)	28 (8.48%)	−0.011	22 (8.80%)	23 (9.20%)	−0.014
Opioid use disorder	3 (0.91%)	2 (0.61%)	0.032	2 (0.80%)	1 (0.40%)	0.046
*Staphylococcus*	51 (15.45%)	49 (14.85%)	0.017	58 (23.20%)	54 (21.60%)	0.038
*Streptococcus*	87 (26.36%)	75 (22.73%)	0.083	68 (27.20%)	70 (28.00%)	−0.018
*Enterococcus*	40 (12.12%)	43 (13.03%)	−0.028	29 (11.60%)	32 (12.80%)	−0.038
Gram-negative bacilli	15 (4.55%)	16 (4.85%)	−0.014	15 (6.00%)	14 (5.60%)	0.017
Fungus	3 (0.91%)	1 (0.30%)	0.064			0.000

*mAVR*, Mechanical aortic valve replacement; *bAVR*, bioprosthetic aortic valve replacement; *SMD*, standardized mean difference; *mMVR*, mechanical mitral valve replacement; *bMVR*, bioprosthetic mitral valve replacement; *SD*, standard deviation.

**Figure 4:**
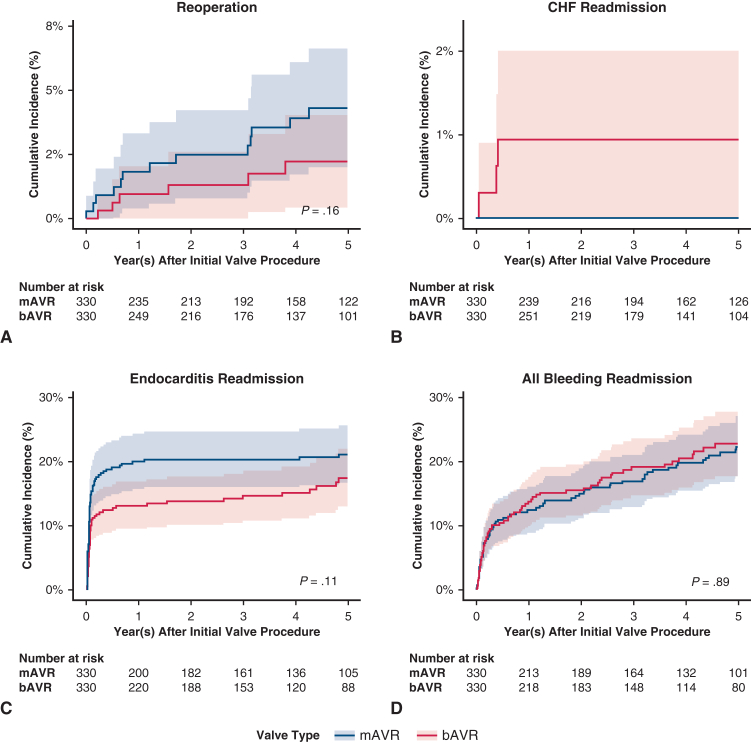

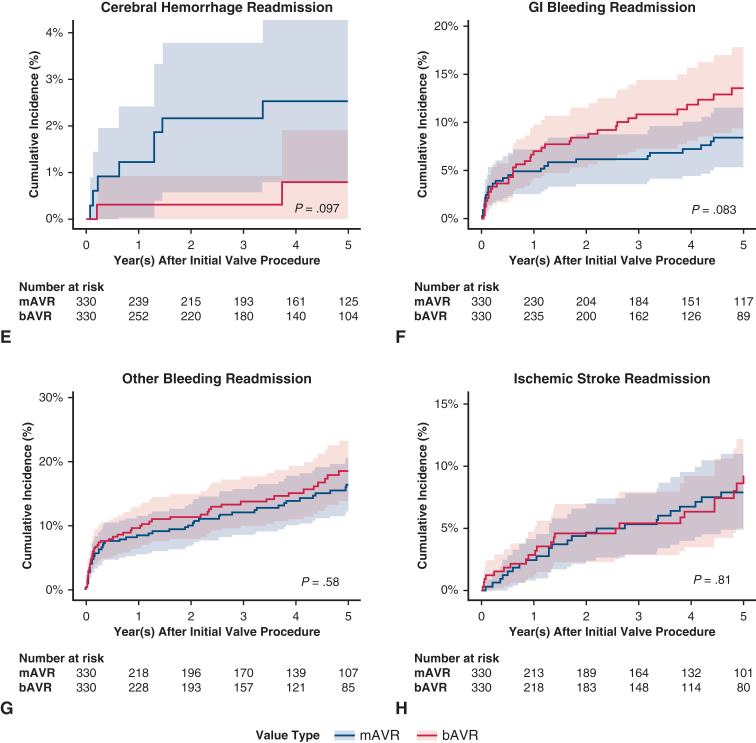
A-H, Secondary outcomes cumulative incidence functions, bAVR versus mAVR with 95% CI. *bAVR*, Bioprosthetic aortic valve replacement; *mAVR*, mechanical aortic valve replacement; *CI*, confidence interval; *CHF*, congestive heart failure; *GI*, gastrointestinal.

Figure 4, *A-H*, show the cumulative incidence curves for the long-term secondary outcomes; Table 2 shows the cumulative incidences of secondary outcomes at 5 years. Compared with bAVR at 5 years of follow-up, patients who underwent mAVR had similar rates of reoperation, congestive heart failure (CHF) readmission, overall bleeding events, and ischemic stroke; there was a slightly greater rate of cerebral hemorrhage among mAVR; however, this did not reach statistical significance. Table E3 shows the subhazard ratios with 95% CIs.

**Table 2:** Secondary outcomes: Cumulative incidence at 5 years

Outcome	mAVR	bAVR	*P*	mMVR	bMVR	*P*
Reoperation	0.031	0.016	.182	0.009	0.028	.154
CHF readmission	0	0.009	−	−	0.008	−
Infective endocarditis readmission	0.178	0.142	.177	0.205	0.195	.768
Composite bleeding readmission	0.183	0.182	.966	0.240	0.213	.490
Cerebral hemorrhage readmission	0.032	0.010	.090	0.026	0.023	.846
Gastrointestinal bleeding readmission	0.078	0.115	.106	0.138	0.101	.199
Other bleeding readmission	0.139	0.152	.628	0.208	0.192	.624
Ischemic stroke readmission	0.076	0.081	.804	0.104	0.073	.233

*mAVR*, Mechanical aortic valve replacement; *bAVR*, bioprosthetic aortic valve replacement; *mMVR*, mechanical mitral valve replacement; *bMVR*, bioprosthetic mitral valve replacement.

### bMVR Versus mMVR Matching and Outcomes

Figure 2 shows the standardized mean differences among matched covariates, indicating a well-balanced match. Matching yielded 250 patients in each group. Table 1 shows cohort characteristics after matching. Five-year overall survival was similar at 53.1% (Figure 3) with RMST at 3.35 years for mMVR and 3.50 years for bMVR with a RMST difference of 0.15 (95% CI, −0.230 to 0.516; *P* = .451).

**Figure 5:**
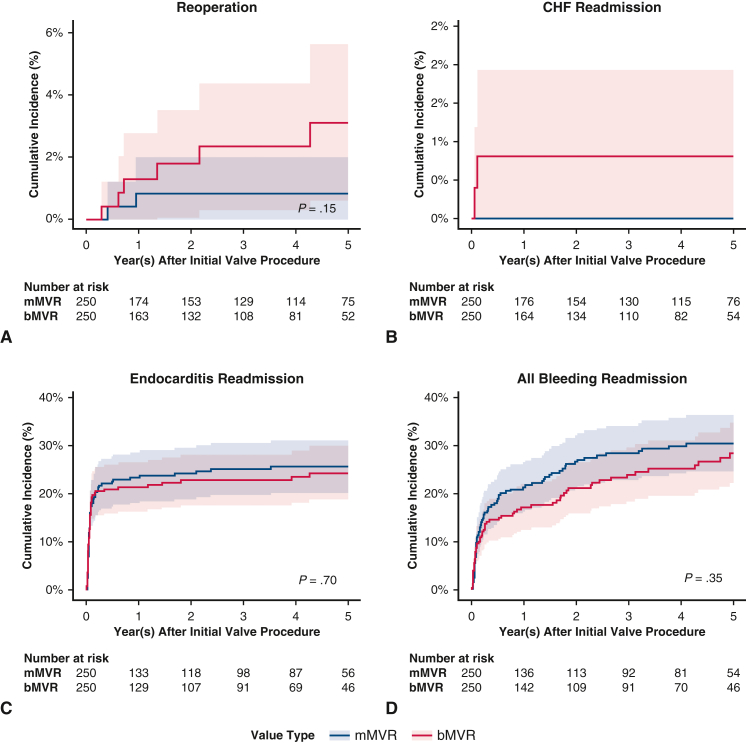

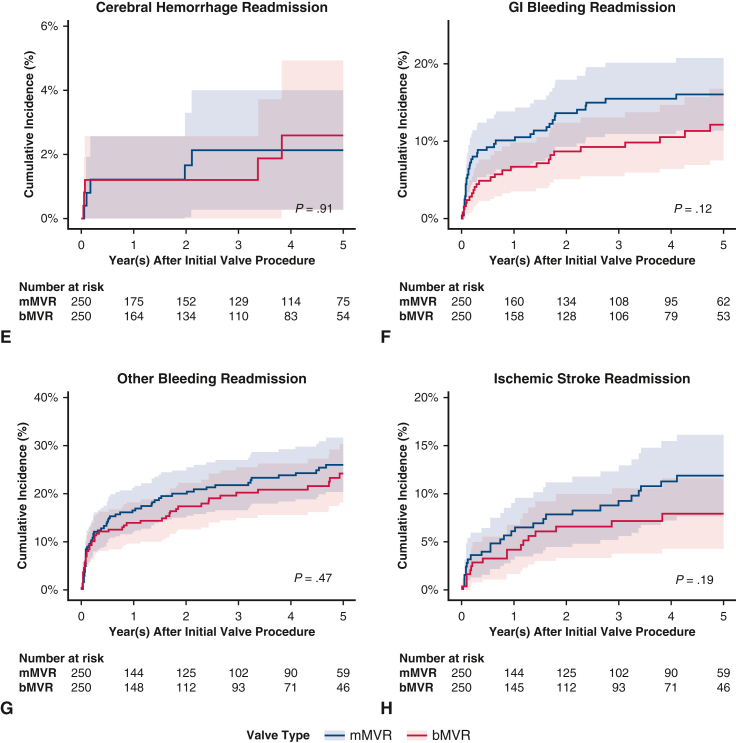
A-H, Secondary outcomes cumulative incidence functions, bMVR versus mMVR with 95% CI. *bMSVR*, Bioprosthetic mitral valve replacement; *mMVR*, mechanical mitral valve replacement; *CI*, confidence interval; *CHF*, congestive heart failure; *GI*, gastrointestinal.

Figure 5, *A-H*, show the cumulative incidence curves for the long-term secondary outcomes; Table 2 shows the cumulative incidences of secondary outcomes at 5 years. Compared with bMVR, at 5 years of follow-up, patients who had undergone mMVR had similar rates of reoperation, CHF readmission, overall bleeding events, and ischemic stroke; compared with bMVR, there was a lower rate of reoperation and greater rate of gastrointestinal bleeding among mAVR; however, these did not reach statistical significance. Table E4 shows the subhazard ratios with 95% CIs.

### Falsification End-Point Analysis

**Figure E2:**
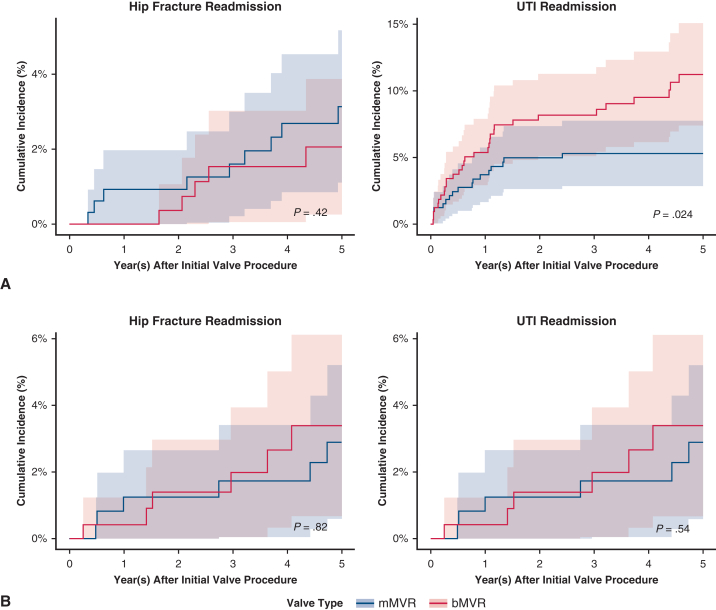
A and B, Falsification end points among matched cohorts with 95% confidence interval. *mAVR*, Mechanical aortic valve replacement; *bAVR*, bioprosthetic aortic valve replacement; *mMVR*, mechanical mitral valve replacement; *bMVR*, bioprosthetic mitral valve replacement.

To increase the robustness of our analysis and assess for unmeasured confounding after matching, the cumulative incidences of UTI and hip fracture were compared among matched groups. As shown in Figure E2, *A*, at the aortic position there were no difference in the cumulative incidences of hip fracture (*P* = .417) but a greater incidence of UTI among bAVR compared with mAVR (*P* = .025). Meanwhile, there was no difference in the incidences of hip fracture (*P* = .822) or UTI (*P* = .539) between mMVR compared with bMVR, as shown in Figure E2, *B*.

### Subgroup Analysis of Patients With Part D Data, 2016-2019

Of 1386 patients from 2016 to 2019 who met all inclusion and exclusion criteria, 915 patients also had Part D Medicare files available for review. Among these 915 patients, 246 patients (26.9%) had filled an anticoagulation prescription within 6 months of surgery. There was no difference in the rates of mechanical versus bioprosthetic valve choice between patients with preoperative anticoagulation and those with no anticoagulation (Table E5).

To evaluate balance of the main indications for preoperative anticoagulation in our entire matched cohort, we compared the rates of preoperative atrial fibrillation and venous thromboembolism (pulmonary embolism or deep venous thrombosis) in our matched cohorts and found no significant differences in rates of atrial fibrillation or venous thromboembolism between mechanical or bioprosthetic valves choices (Table E6).

### Subgroup Analysis of Excluded Patients With Preoperative Ischemic or Hemorrhagic Stroke

In total, 613 patients were excluded because of a preoperative ischemic or hemorrhagic stroke within 30 days before surgery; compared with patients with no preoperative stroke, there was no difference in prosthesis choice type (Table E7).

### Subgroup Analysis by Age Subgroups

**Figure E3:**
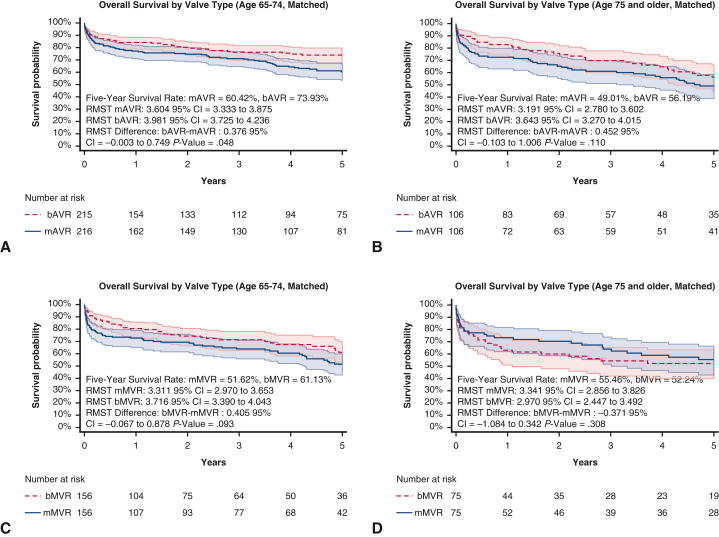
Overall survival by matched age subgroups with 95% confidence interval. A, Aortic valve replacement in patients 65 to 74 years old. B, Aortic valve replacement in patients 75 and greater. C, Mitral valve replacement in patients 65 to 74 years old. D, Mitral valve replacement in patients 75 and greater. *RMST*, Restricted mean survival time; *mAVR*, mechanical aortic valve replacement; *CI*, confidence interval; *bAVR*, bioprosthetic aortic valve replacement; *mMVR*, mechanical mitral valve replacement; *bMVR*, bioprosthetic mitral valve replacement.

We repeated our analysis stratifying our overall cohort into 2 age subgroups: age 65 to 74 years old and age ≥75 years. We performed separate propensity score matching in each age subgroup at the aortic and mitral positions. For patients 65 to 74 years old, mAVR was associated with a worse 5-year overall survival compared with bAVR with an RMST difference of 0.38 years (95% CI, 0.003-0.75, *P* = .04) and mMVR was not significantly associated with a difference in 5-year overall survival compared with bMVR, with an RMST difference of 0.41 (95% CI, −0.07 to 0.88, *P* = .093). For patients ≥75 years, mAVR was not significantly associated with a worse 5-year overall survival compared with bAVR, with an RMST difference of 0.45 years (95% CI, −0.10 to 1.0, *P* = .110) and mMVR was not significantly associated with a difference in 5-year overall survival compared with bMVR, with an RMST difference of −0.37 (95% CI, −1.08 to 0.34, *P* = .308). Figure E3, *A-D*, shows the primary outcome analysis by age subgroups.

### Post Hoc Logistic Regression Identifying Predictors of 30-Day and 90-Day Mortality

Both 30-day and 90-day mortality were significantly greater among mechanical valve replacements for both AVR and MVR (Table E8). For AVR, 30-day mortalities were 12.4% and 5.5%, whereas 90-day mortalities were 18.5% and 8.5% for mAVR and bAVR, respectively. For MVR, 30-day mortalities were 15.6% and 8.4%, whereas 90-day mortalities were 22.0% and 12.8% for mMVR and bMVR, respectively. On multivariable logistic regression modeling, compared with mechanical valves, bioprosthetic valves were significantly associated with a lower odds ratio (OR) of 30-day mortality (bAVR OR, 0.34; 95% CI, 0.18-0.67; bMVR OR, 0.41; 95% CI, 0.22-0.78) and 90-day mortality (bAVR OR, 0.41; 95% CI, 0.24-0.68; bMVR OR, 0.43; 95% CI, 0.25-0.73). Table E9 shows the multivariable models.

### Incidence of Fatal Bleeding by Valve Type

**Figure E4:**
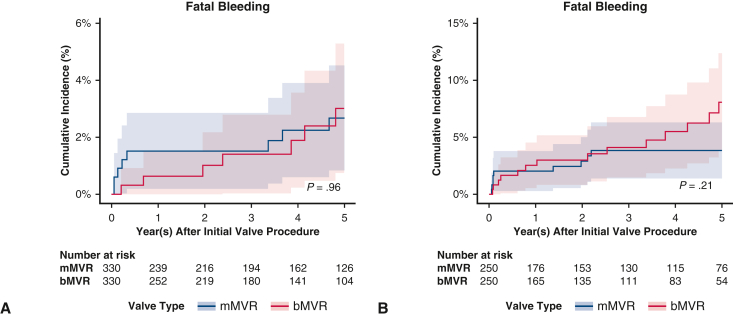
A and B, Cumulative incidence of fatal bleeding with 95% confidence interval. A, Aortic valve replacement. B, Mitral valve replacement. *mAVR*, Mechanical aortic valve replacement; *bAVR*, bioprosthetic aortic valve replacement; *mMVR*, mechanical mitral valve replacement; *bMVR*, bioprosthetic mitral valve replacement.

To examine the increased early mortality associated with mechanical valve choice, we examined the incidence of readmissions for bleeding complications that resulted in mortality after AVR and MVR. We found no difference in fatal bleeding events by valve choice at the aortic (*P* = .961) and mitral (*P* = .214) positions, as shown in Figure E4, *A* and *B*.

## Discussion

In this retrospective review of Medicare data, we compared propensity score−matched cohorts of adults >65 years old who underwent either bioprosthetic or mechanical valve replacement for active left-sided endocarditis, stratifying our comparison by the aortic and mitral position. Patients with preoperative stroke were excluded to prevent treatment bias, because mandatory anticoagulation in these patients is not recommended. Before matching, nearly 21% of patients received a mechanical valve, whereas 79% received a bioprosthetic valve. Our matched analysis found that patients undergoing mAVR had worse associated short-term 30-day and 90-day survival and 5-year overall survival compared with bAVR. Similarly, there was significantly decreased associated early survival for mMVR as compared with the bMVR but no difference in 5-year overall survival. In addition, there were no major significant differences between valve types for several secondary outcomes including reoperation, CHF readmission, bleeding events, and ischemic stroke. These findings add to the paucity of data examining valve prosthesis choice in patients >65 years afflicted with left-sided endocarditis in the largest cohort to date.

Most recent well-sized cohort studies comparing prosthesis choice for left-sided IE have analyzed survival among patients <65 years old. In a 2023 study using data from the Italian INFECT-Registry, Salsano and colleagues^5^ found improved survival and decreased IE recurrence associated with mAVR over bAVR in 549 patients aged 45 to 65 years with aortic valve IE. In 2020, Hu and colleagues^3^ published a cohort of 492 matched patients aged 50 to 69 years in the Hubei province hospitals and found improved survival associated with mMVR over bMVR with 15 years of follow-up data. Contrary to these findings, a 2018 study using California and New York state data, Toyoda and colleagues^15^ analyzed 3447 patients with left-sided IE and found no difference in all-cause survival and reinfection between bioprosthetic and mechanical prosthesis choice; although their analysis was not restricted to younger patients, the mean ages were 61.4 years and 54.0 years for bioprosthetic and mechanical valves, respectively. In 2021, Formica and colleagues^16^ reported the largest meta-analysis comparing prosthesis type for IE, compiling data from 13 studies including the 2018 study by Toyoda and colleagues.^15^ They found that in 8285 total patients with a pooled mean age of 55.1 years, the use of mechanical prosthesis was associated with improved survival and reoperation rates compared with bioprosthetic prosthesis for IE. Taken collectively, these studies provide a convincing portfolio of data that in middle-aged patients with left-sided IE, mechanical valves provide a survival benefit over bioprosthetic valves.

Meanwhile there are limited data in patients >65 years with IE. Two previous studies have compared prosthesis choice directly among older patients. Nguyen and colleagues,^6^ using national data in France, demonstrated that bAVR was associated with a lower 5-year overall survival compared with mAVR in patients <65 years old; however, they did not find any significant difference in survival in patients >65 years old; notably, their cohort contained only 47 patients total who were >65 years. In a more recent single-institution study, Kahrovic and colleagues^4^ found no difference in overall survival or secondary outcomes between prosthesis choices for patients with left-sided IE in 132 patients >55 years old. Although smaller cohort sizes, these studies do show similar findings to our report, which demonstrates that at minimum the survival benefit of mechanical valves is not noted in patients >65 years. In fact, our study suggests worse survival of mechanical valves for IE in the aortic position. Considering that surgical intervention in older patients with IE has been reported to be both underused^17^ yet beneficial in terms of survival,^17^, ^18^, ^19^ our findings are important to help physicians guide acutely ill patients >65 years through their perioperative treatment course.

Fitting our study into the broader context of prosthetic choice for AVR and MVR for all surgical indications, 2 large American cohort studies with long-term follow-up have now been published comparing bioprosthetic and mechanical valve choice for AVR and MVR. These 2 studies concluded that mechanical prostheses are associated with improved survival in younger patients; however, that survival benefit does not persist for older patients. Specifically, Goldstone and colleagues,^20^ in a cohort of patients from California, demonstrated a survival benefit associated with mechanical over bioprosthetic prosthesis in patients up to age 55 years for AVR and age 70 years for MVR. Similarly, Bowdish and colleagues^21^ analyzed a large cohort of patients undergoing AVR from the Society of Thoracic Surgeons adult cardiac surgery database and found a survival benefit in patients up to 60 years associated with mechanical prosthesis and survival equipoise after age 60 years. Our study in older patients, as well as the other studies discussed in younger patients, corroborate these findings among the subgroup of patients undergoing AVR and MVR who have IE. Curiously, however, for AVR our findings not only demonstrate no benefit to mechanical valves but rather a worse survival associated with mechanical valves. Although there are possible unmeasured confounders driving this result, these findings are nonetheless surprising in that one would expect that any residual unmeasured confounding would favor mAVR because at baseline these patients were younger with fewer comorbidities. What is also surprising is the frequency with which mechanical valves were used in our cohort at 20.9% of patients, especially when guidelines give a class 2A recommendation for bioprosthetic valves over mechanical valves in patients >65 years old.^8^ Thus, one can assume that surgeons would be more selective for patients they believed would live long enough to reap the benefit of a more durable valve while also inheriting the increased bleeding complications and lifestyle burden associated with mechanical valves.^20^ In an attempt to assess the adequacy of our propensity score matching to balance unmeasured confounders that may have impacted our results, we performed a falsification end-point analysis comparing the incidences of UTI and hip fracture among our propensity-matched cohorts. At the mitral position, we found no significant differences in either UTIs or hip fracture, indicating a balanced match. At the aortic position, we found no difference in the rates of hip fractures but a greater incidence of UTI among bAVR compared with mAVR, thus indicating that there may have been some residual unmeasured confounding in our AVR analysis. Interestingly, however, again one would expect the opposite trend with patients undergoing mAVR having greater risk of UTI, given their greater risk of postoperative mortality, rather than our finding of greater UTI among patients undergoing bAVR. Thus, we believe our propensity-matched analyses were overall well-matched and that the mortality difference at the AVR position with worse mortality for mAVR was apparent even with potential confounding in favor of mAVR. It remains unclear from our analysis why this survival difference was only found for AVR but not MVR; the similarities in secondary outcomes in our study do not provide much insight or explanation, either.

Certainly, there are limitations to our study. Claims databases lack the granularity needed to account for all potentially confounding variables. We do believed that our statistical methods used provide the best attempt to account for measurable confounding variables available in the Medicare MEDPAR database. In addition, we excluded patients with ischemic and hemorrhagic stroke within 1 month of surgery to reduce confounding by indication. It should be noted, however, that when we analyzed the subgroups of patients who had a preoperative stroke or preoperative anticoagulation, there were no differences in valve choices. There is the separate issue of ICD coding errors and misclassification bias, which cannot be mitigated with the use of Medicare data and tends to bias hypothesis testing towards the null. Other unmeasured factors may also impact surgeon prosthesis choice. For example, surgeons may choose a mechanical valve to avoid patient-prosthesis mismatch; however, there are no data regarding body surface area or valve size implanted to make any inferences about this possibility. Given the high early mortality rates associated with mechanical valves, a tradeoff of patient-prosthesis mismatch for a lower mortality with a bioprosthetic valve may be a consideration in this cohort. The MEDPAR database does not provide data about cause of death, which limits our ability to explain why mechanical valves were associated with great early mortality. We did compare rates of fatal bleeding in an attempt to explain this early divergence in mortality; however, we found no difference in bleeding mortality at the aortic or mitral position. Thus, it remains unclear in our study why early mortality was greater with mechanical valve choice. Lastly, although our cohort is relatively contemporary, this study is limited to only 5 years of follow-up, and longer-term follow-up is still needed.

## Conclusions

Patients >65 years old can undergo valve replacement for left-sided endocarditis with reasonable 5-year outcomes. We found low incidences of postoperative complications such as reinfections, readmissions, and stroke. However, this study also suggests that in this population, there is no associated benefit of mechanical valve choice in the aortic or mitral position, regardless of a patient's preoperative need for long-term anticoagulation. An important finding considering that nearly 21% of patients received a mechanical valve. In contrast to previous studies comparing outcomes between mechanical and bioprosthetic valve choices for younger patients with left-sided IE, our evaluation of older patients >65 years old demonstrated that mechanical valves conferred no 5-year survival advantage over bioprosthetic valves and may be associated with a worse survival in the aortic position.

### Webcast

You can watch a Webcast of this AATS meeting presentation by going to: https://www.aats.org/resources/cardiac-valve-prosthesis-choic-9952.

**Figure:**
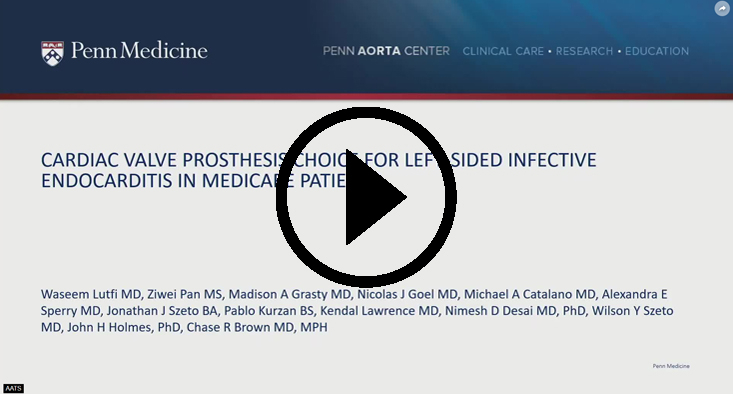

### Audio

You can listen to the discussion audio of this article by going to the supplementary material section below.

## Conflict of Interest Statement

Dr Desai has consulted for W. L. Gore & Associates and Terumo Aortic, which had no influence on the study design or conclusions. Dr Szeto has consulted for Abbott, Artivion, Edwards Lifesciences, Medtronic, and Terumo Aortic, for whom he is an investigator, advisory board, and speaker. All other authors reported no conflicts of interest.

The *Journal* policy requires editors and reviewers to disclose conflicts of interest and to decline handling or reviewing manuscripts for which they may have a conflict of interest. The editors and reviewers of this article have no conflicts of interest.

## Figures and Tables

**Table E1:** ICD coding schema

Infective endocarditis (IE)	ICD-9-CM: 421, 4210, 4219, 4211, 4249, 42499, 42490, 42491, 11281, 03642, 09884, 11404, 11515, 11594 ICD-10-CM: 133, I330, I339, I38, I39, A3951, B376
AVR type	
mAVR	ICD-9-PCS: 3522 ICD-10-PCS: 02RF0JZ
bAVR	ICD-9-PCS: 3521 ICD-10-PCS: 02RF07Z, 02RF08Z, 02RF0KZ
MVR type	
mMVR	ICD-9-PCS: 3524 ICD-10-PCS: 02RG0JZ
bMVR	ICD-9-PCS: 3523 ICD-10-PCS: 02RG07Z, 02RG08Z, 02RG0KZ
Other valve procedures	
Tricuspid valve replacement	ICD-9-PCS: 3527, 3528 ICD-10-PCS: 02RJ07Z, 02RJ08Z, 02RJ0KZ, 02RJ0JZ
Aortic valve repair	ICD-9-PCS: 3511 ICD-10-PCS: 02QF
Mitral valve repair	ICD-9-PCS: 3512 ICD-10-PCS: 02QG0
Transcatheter mitral valve repair	ICD-9-PCS: N/A ICD-10-PCS: 02RG3
Pulmonary valve repair	ICD-9-PCS: 3513 ICD-10-PCS: 02QH
Tricuspid valve repair	ICD-9-PCS: 3514 ICD-10-PCS: 02WJ
Transcatheter aortic valve replacement	ICD-9-PCS: 3505, 3506 ICD-10-PCS: 02RF3, 02RF4
Pulmonary valve replacement	ICD-9-PCS: 3507, 3508, 3525, 3526 ICD-10-PCS: 02RH
Open thoracic aortic procedure	ICD-9-PCS: 3804, 3814, 3834, 3845, 3865 ICD-10-PCS: 02RX, 02RW, 02QX, 02QW, 02VX, 02VW, 02HX, 02HW
Concomitant procedures	
CABG	ICD-9-PCS: 361, 362, 363 ICD-10-PCS: 0210, 0211, 0212, 0213
PCI	ICD-9-PCS: 0066, 1755, 3601, 3602, 3603, 3605, 3606, 3607, 3609, 3633, 3634 ICD-10-PCS: 0270, 0271, 0272, 0273, 02H0, 02H1, 02H2, 02H3
Heart transplant or ventricular assist device	ICD-9-PCS: 336, 3751, 3752, 375, 3753, 3754, 3755, 3760, 3762, 3763, 3765, 3766, 3768 ICD-10-PCS: 02HA0QZ, 02HA0RJ, 02HA0RS, 02HA0RZ, 02HA3QZ, 02HA3RJ, 02HA3RS, 02HA3RZ, 02HA4QZ, 02HA4RJ, 02HA4RS, 02HA4RZ, 02RK07Z, 02RK08Z, 02RK0JZ, 02RK0KZ, 02RK47, 02RK47Z, 02RK48Z, 02RK4JZ, 02RK4KZ, 02RL07Z, 02RL08Z, 02RL0JZ, 02RL0KZ, 02RL47Z, 02RL48Z, 02RL4JZ, 02RL4KZ, 02UA0JZ, 02UA3JZ, 02UA4JZ, 02WA0JZ, 02WA0QZ, 02WA3QZ, 02YA0Z0, 02YA0Z1, 02YA0Z2, 5A02116, 5A0211D
Other outcomes	
Cerebral hemorrhage	ICD-9-DM: 430-432 ICD-10-DM: I60, I61, I62
Ischemic stroke	ICD-9-DM: 433, 434, 436, 4371, 4378, 4379 ICD-10-DM: I63, I64, I65, I66, I6781, I6782, I6789, I679
GI hemorrhage	ICD9-DM: 4552, 4555, 4558, 4560, 45620, 5307, 53082, 5310- 5316, 5320-5326, 5330-5336, 5340-5346, 53501-53561, 56202-56203, 56212-56213, 5693, 56985, 5780, 5781, 5789, 53783, 56881 ICD10-DM: I8501, I8511, K644, K648, K226, K228, K250-K256, K260- K266, K270-K276, K280-K286, K2901, K2911, K2921, K2931, K2941, K2951, K2961, K2971, K2981, K2991 K5701, K5711, K5713, K5721, K5731, K5733, K5741, K5751, K5753, K5781, K5791, K5793, K625, K5521, K920, K921, K922, K31811, K661
Other bleeding	ICD-9-DM: 2851, 28659, 42989, 4230, 459, 59970, 59971, 59972, 71911, 7847, 7848, 7863, 852, 853, 9901, 9904, 99702, 9981, 9998 ICD-10-DM: D62, D68311, D68312, D68318, I312, I513, I9741, I9742, I9761, K920, M25019, R042, R040, R041, R042, R0481, R0489, R049, R310, R311, R312, R319, R58, S064X3A, S064X7A, S065X1A, S065X5A, S065X9A, S066X3A, S066X7A, S064X0A, S064X1A, S064X4A, S064X5A, S064X8A, S064X9A, S065X2A, S065X3A, S065X6A, S065X7A, S066X0A, S066X1A, S066X4A, S066X5A, S066X8A, S066X9A. S064X2A, S064X6A, S065X0A, S065X4A, S065X8A, S066X2A, S066X6
Congestive heart failure	ICD-9-DM: 39891, 428, 4280, 4281, 4282, 42820, 42821, 42822, 42823, 4283, 42830, 42831, 42832, 42833, 4284, 42840, 42841, 42842, 42843, 4289, 429, 4290, 4291, 4292, 4293, 4294, 4295, 4296, 4297, 42971, 42979, 4298, 42981, 42982, 42983, 42989, 4299 ICD-10-DM: I0981, I110, I130, I132, I501, I5020, I5021, I5022, I5023, I5030, I5031, I5032, I5033, I5040, I5041, I5042, I5043, I50810, I50811, I50812, I50813, I50814, I5082, I5083, I5084, I5089, I509 , I5181, I97110, I97130, I97190
Micro-organisms	
*Staphylococcus*	ICD-9-CM: 0381, 03810, 03811, 03812, 03819, 0411, 04110, 04111, 04112, 04119 ICD-10-CM: A410, A4101, A4102, A4901, A4902, B956, B9561, B9562, B957, B958, J15211, J15212
*Streptococcus*	ICD-9-CM: 0380, 0382, 0410, 04100, 04101, 04102, 04103, 04105, 04109, 0412 ICD-10-CM: A400, A401, A403, A408, A409, A491, B950, B951, B953, B954, B955
*Enterococcus*	ICD-9-CM: 04104 ICD-10-CM: B952, A4181
Gram-negative bacilli	ICD-9-CM: 0384, 03840, 03841, 03842, 03843, 03844, 03849, 0413, 0414, 0415, 0416, 0417, 04185 ICD-10-CM: A413, A4150, A4151, A4152, A4153, A4159, B961, B9620, B9621, B9622, B9623, B9629, B963, B964, B965
Fungus	ICD-9-CM: 1125, 11281, 1160, 11504, 11514, 11594, 1173 ICD-10-CM: B376, B377, B409, B393, B394, B395, B399, B449
IE risk factors	
Prosthetic valve	ICD-9-DM: V433, V422 ICD-10-DM: Z952
Cardiac devices	ICD-9-CM: V4501, V5331, V4502, V5332, 99601, 99604 ICD-10-CM: Z950, Z9581
Opioid use disorder	ICD-9-CM: 3040, 3055 ICD-10-CM: F11
Match balance confirmation	
Atrial fibrillation	ICD-9-DM: 4273 ICD-10-DM: I48
Venous thromboembolism	ICD-9-DM: 4151, 451 ICD-10-DM: I26, I824, I825, I826, I827

*ICD*, *International Classification of Diseases*; *AVR*, aortic valve replacement; *mAVR*, mechanical aortic valve replacement; *PCS*, Procedure Coding System; *bAVR*, bioprosthetic aortic valve replacement; *MVR*, mitral valve replacement; *mMVR*, mechanical mitral valve replacement; *bMVR*, bioprosthetic mitral valve replacement; *CABG*, coronary artery bypass grafting; *PCI*, percutaneous coronary intervention; *GI*, gastrointestinal.

**Table E2:** Unmatched cohort characteristics

Characteristic	mAVR	bAVR	SMD	mMVR	bMVR	SMD
N	334	1386		257	852	
Age, y, mean (SD)	72.96 (5.62)	73.56 (5.65)	−0.107	72.71 (5.15)	73.46 (5.49)	−0.146
Sex			0.147			−0.035
Male	236 (70.66%)	1072 (77.34%)		142 (55.25%)	456 (53.52%)	
Female	98 (29.34%)	314 (22.66%)		115 (44.75%)	396 (46.48%)	
Elixhauser score, mean (SD)	7.09 (2.94)	7.92 (3.04)	−0.283	7.47 (3.01)	7.73 (3.20)	−0.086
Coronary artery bypass graft	69 (20.66%)	140 (10.10%)	0.261	40 (15.56%)	70 (8.22%)	0.203
Percutaneous coronary intervention	8 (2.40%)	14 (1.01%)	0.091	0 (0.00%)	4 (0.47%)	0.059
Prosthetic valve	70 (20.96%)	282 (20.35%)	0.015	44 (17.12%)	121 (14.20%)	0.077
Cardiac device	27 (8.08%)	128 (9.24%)	−0.042	22 (8.56%)	88 (10.33%)	−0.063
Opioid use disorder	3 (0.90%)	12 (0.87%)	0.003	2 (0.78%)	12 (1.41%)	−0.072
*Staphylococcus*	53 (15.87%)	235 (16.96%)	−0.030	58 (22.57%)	216 (25.35%)	−0.067
*Streptococcus*	88 (26.35%)	338 (24.39%)	0.045	73 (28.40%)	254 (29.81%)	−0.031
*Enterococcus*	40 (11.98%)	249 (17.97%)	−0.185	29 (11.28%)	117 (13.73%)	−0.077
Gram-negative bacillis	16 (4.79%)	65 (4.69%)	0.005	15 (5.84%)	47 (5.52%)	0.014
Fungus	3 (0.90%)	14 (1.01%)	−0.012	1 (0.39%)	7 (0.82%)	−0.063

*mAVR*, Mechanical aortic valve replacement; *bAVR*, bioprosthetic aortic valve replacement; *SMD*, standardized mean difference; *mMVR*, mechanical mitral valve replacement; *bMVR*, bioprosthetic mitral valve replacement; *SD*, standard deviation.

**Table E3:** Subhazard regression (SHR) with death as a competing risk, mAVR (reference) versus bAVR

Outcomes	SHR	95% CI	*P* value
Reoperation	0.518	0.197-1.362	.182
CHF readmission	−	−	−
Infective endocarditis readmission	0.780	0.544-1.119	.177
Composite bleeding readmission	0.993	0.709-1.389	.966
Cerebral hemorrhage readmission	0.324	0.088-0.192	.090
Gastrointestinal bleeding readmission	1.516	0.915-2.510	.106
Other bleeding readmission	1.101	0.746-1.626	.628
Ischemic stroke readmission	1.075	0.606-1.906	.804

*mAVR*, Mechanical aortic valve replacement; *bAVR*, bioprosthetic aortic valve replacement; *CI*, confidence interval; *CHF*, congestive heart failure.

**Table E4:** Subhazard regression (SHR) with death as a competing risk, mMVR (reference) versus bMVR

Outcomes	SHR	95% CI	*P* value
Reoperation	3.238	0.644-16.28	.154
CHF readmission	−	−	−
Infective endocarditis readmission	0.948	0.664-1.353	.768
Composite bleeding readmission	0.887	0.632-1.245	.490
Cerebral hemorrhage readmission	0.889	0.269-2.936	.846
Gastrointestinal bleeding readmission	0.178	0.433-1.190	.199
Other bleeding readmission	0.911	0.629-1.321	.624
Ischemic stroke readmission	0.692	0.378-1.268	.233

*mMVR*, Mechanical mitral valve replacement; *bMVR*, bioprosthetic mitral valve replacement; *CI*, confidence interval; *CHF*, congestive heart failure.

**Table E5:** Valve prosthesis choice by preoperative anticoagulation status among patients with Part D data, 2016-2019

Valve choice	Anticoagulation	No anticoagulation	*P* value
AVR			
mAVR	12 (7.2%)	24 (6.1%)	.623
bAVR	155 (92.8%)	371 (93.9%)	
MVR			
mMVR	11 (13.9%)	38 (13.9%)	.990
bMVR	68 (86.1%)	236 (86.1%)	
Total			
Mechanical	23 (9.3%)	62 (9.3%)	.970
Bioprosthetic	223 (90.7%)	607 (90.7%)	

*AVR*, Aortic valve replacement; *mAVR*, mechanical aortic valve replacement; *bAVR*, bioprosthetic aortic valve replacement; *MVR*, mitral valve replacement; *mMVR*, mechanical mitral valve replacement; *bMVR*, bioprosthetic mitral valve replacement.

**Table E6:** Balance of atrial fibrillation and venous thromboembolism in the matched cohorts

Outcome	mAVR	bAVR	SMD	mMVR	bMVR	SMD
n	330	330		250	250	
Atrial fibrillation	70 (21.2%)	81 (24.6%)	0.079	62 (24.8%)	73 (29.2%)	0.099
Venous thromboembolism	7 (2.1%)	8 (2.4%)	0.020	12 (4.8%)	9 (3.6%)	0.060

*mAVR*, Mechanical aortic valve replacement; *bAVR*, bioprosthetic aortic valve replacement; *SMD*, standardized mean difference; *mMVR*, mechanical mitral valve replacement; *bMVR*, bioprosthetic mitral valve replacement.

**Table E7:** Valve prosthesis choice by preoperative ischemic or hemorrhagic stroke status

Valve choice	Stroke	No stroke	*P* value
AVR			
mAVR	58 (18.2%)	334 (19.4%)	.623
bAVR	260 (81.8%)	1386 (80.6%)	
MVR			
mMVR	56 (19.0%)	257 (23.2%)	.124
bMVR	239 (81.0%)	852 (76.8%)	
Total			
Mechanical	114 (18.6%)	591 (20.1%)	.203
Bioprosthetic	499 (81.4%)	2238 (79.9%)	

*AVR*, Aortic valve replacement; *mAVR*, mechanical aortic valve replacement; *bAVR*, bioprosthetic aortic valve replacement; *MVR*, mitral valve replacement; *mMVR*, mechanical mitral valve replacement; *bMVR*, bioprosthetic mitral valve replacement.

**Table E8:** 30-day and 90-day mortality among matched cohorts

Mortality	mAVR	bAVR	mMVR	bMVR
n	330	330	250	250
30-d mortality	41 (12.4%)	18 (5.5%)	39 (15.6%)	21 (8.4%)
90-d mortality	61 (18.5%)	28 (8.5%)	55 (22.0%)	32 (12.8%)

*mAVR*, Mechanical aortic valve replacement; *bAVR*, bioprosthetic aortic valve replacement; *mMVR*, mechanical mitral valve replacement; *bMVR*, bioprosthetic mitral valve replacement.

**Table E9:** Forward stepwise logistic regression models identifying predictors of 30-day and 90-day mortality among matched cohorts

Predictors	Odds ratio (95% confidence interval)	*P* value
AVR 30-d mortality: bAVR vs mAVR (reference)		
bAVR	0.35 (0.18-0.67)	.002
Age, y	1.03 (0.98-1.09)	.256
Female	0.79 (0.39-1.62)	.523
Complicated diabetes	2.04 (0.89-4.67)	.091
Weight loss	0.54 (0.26-1.13)	.104
Paralysis	6.06 (1.97-18.62)	.002
Deficiency anemia	2.29 (0.97-5.42)	.059
Prosthetic valve	2.40 (1.23-4.65)	.009
Obesity	0.07 (0.01-0.78)	.030
Electrolyte disorders	1.84 (0.86-3.91)	.115
Liver disease	6.24 (2.54-15.33)	<.001
Renal failure	2.28 (1.19-4.39)	.013
Metastatic cancer	5.00 (0.82-30.5)	.081
Solid tumor	0.23 (0.05-1.07)	.061
Other neurologic disorders	2.11 (1.06-4.24)	.034
Peripheral vascular disease	1.87 (0.95-3.68)	.070
AVR 90-d mortality: bAVR vs mAVR (reference)		
bAVR	0.41 (0.24-0.68)	.001
Age, y	1.02 (0.97-1.06)	.469
Female	0.92 (0.52-1.63)	.786
Peripheral vascular disease	1.85 (1.06-3.23)	.030
Liver disease	2.50 (1.17-5.36)	.019
Renal failure	1.71 (1.02-2.88)	.042
Electrolyte disorders	2.30 (1.22-4.34)	.010
Alcohol use disorders	0.16 (0.07-1.41)	.099
Prosthetic valve	2.28 (1.32-3.94)	.003
Drug use disorder	5.13 (1.06-24.77)	.042
Paralysis	3.48 (1.20-10.04)	.021
Other neurologic disorders	2.46 (1.39-4.34)	.002
Coagulopathy	1.57 (0.94-2.63)	.085
MVR 30-d mortality: bMVR vs mMVR (reference)		
bMVR	0.41 (0.22-0.77)	.006
Age, y	1.01 (0.95-1.07)	.732
Female	1.25 (0.67-2.36)	.477
*Staphylococcus*	2.19 (1.14-4.17)	.017
Hypothyroidism	0.48 (0.21-1.12)	.091
Liver disease	2.62 (1.19-5.75)	.016
Other neurologic disorders	2.01 (1.01-4.00)	.044
Obesity	2.68 (0.74-9.63)	.131
Valvular disease	0.36 (0.17-0.74)	.006
Renal failure	1.65 (0.88-3.09)	.115
Peripheral vascular disease	3.12 (1.54-6.31)	.001
Congestive heart failure	1.99 (0.96-4.12)	.063
Cardiac arrhythmia	0.38 (0.19-0.74)	.005
MVR 90-d mortality: bMVR vs mMVR (reference)		
bMVR	0.43 (0.25-0.73)	.002
Age, y	0.99 (0.95-1.04)	.931
Female	1.34 (0.80-2.26)	.267
*Staphylococcus*	1.68 (0.95-2.99)	.072
Obesity	2.46 (0.89-6.82)	.083
Peripheral vascular disease	3.00 (1.62-5.55)	<.001
Liver disease	2.33 (1.15-4.74)	.019
Valvular disease	0.36 (0.19-0.70)	.002
Weight loss	1.66 (0.94-2.91)	.079
Alcohol use disorder	0.11 (0.02-1.01)	.051
Renal failure	1.82 (1.05-3.12)	.030
Cardiac arrhythmia	0.48 (0.26-0.87)	.017
Other neurologic disorders	1.91 (1.06-3.41)	.029
Congestive heart failure	1.85 (0.99-3.44)	.051

## Supplementary Data

### Audio

Audio 1 This is an audio recording of the presentation of the associated abstract and discussion. The abstract presentation ends at 8:25 and the discussion begins at 8:30.

Video 1 Video available at: https://www.jtcvs.org/article/S2666-2736(25)00382-1/fulltext.
